# Transforming surgical ward rounds: constructivist pedagogy, structured models, and intelligent technologies

**DOI:** 10.3389/fmed.2026.1869574

**Published:** 2026-07-07

**Authors:** Linkun Zhong, Cong Zhou, Jianhang Miao, Jinghang Dong, Lili Luo, Chizhuai Liu, Yutong Li

**Affiliations:** 1 Department of General Surgery, Zhongshan City People’s Hospital, Zhongshan, Guangdong, China; 2 Department of Nephrology, The Third Affiliated Hospital of Guangzhou Medical University, Guangzhou, Guangdong, China; 3 Science and Education Department, Zhongshan City People’s Hospital, Zhongshan, Guangdong, China; 4 The First School of Clinical Medicine, Guangdong Medical University, Zhanjiang, Guangdong, China

**Keywords:** artificial intelligence, BID model, educational, models, SNAPPS, teaching rounds, virtual reality

## Abstract

Surgical ward rounds are central to clinical training but often fail to engage learners as active participants. Traditional apprenticeship models leave teaching unstructured and variable, while clinical pressures compress time available for education. This review examines three interconnected developments that offer a way forward: constructivist learning theories that explain why active engagement matters, structured teaching models that translate theory into practice, and intelligent technologies that expand what is possible. SNAPPS (Summarize, Narrow, Analyze, Probe, Plan, Select), the One-Minute Preceptor (OMP), and the BID (Briefing, Intraoperative, Debriefing) framework each operationalize constructivist principles in different clinical contexts. Preliminary evidence from recent trials suggests that SNAPPS may improve clinical reasoning and case presentation quality, while OMP appears to achieve higher learner satisfaction in time-pressed settings. Generative artificial intelligence (AI) tools, in exploratory studies, can simulate consultant-level questioning before rounds and support consolidation afterwards. Immersive virtual reality (IVR) offers scalable skills training with potential long-term knowledge retention. Yet these technologies introduce concerns about accuracy, over-reliance, and algorithmic bias that governance frameworks are only beginning to address. Drawing on the evidence, an integrated framework is proposed that positions theory as the foundation, structured models as the methodology, and technology as an empowering tool. The field now needs multi-center studies with standardized outcomes, longer follow-up, and systematic investigation of how AI and VR combine with established teaching frameworks.

## 1 Introduction

Teaching on surgical ward rounds is both indispensable and under strain. Rounds offer learners direct exposure to patient care, clinical reasoning, and decision-making under expert supervision (1). At the same time, increasing clinical workloads, shorter hospital stays, and documentation demands have squeezed the time available for teaching (2). The traditional apprenticeship approach, where the learners observe and absorb, too often leaves them as the passive bystanders rather than active participants in knowledge construction (3, 4). Data from a national survey of 148 surgery programs and 998 residents illustrate the gap: while 84% of attending surgeons offered technical advice during operations, only 18% helped the residents identify educational goals beforehand, and just 37% discussed areas for improvement afterwards (5, 6).

Three parallel developments have created new possibilities for addressing these challenges. The first comes from educational theory. Constructivism, both cognitive and social, has gained traction in health professions education, shifting focus from what teachers transmit to what learners build (7, 8). Social constructivism, emphasizes learning as a social process shaped by interaction and guided by more knowledgeable others (9). In the context of ward rounds, this means deliberately designing interactions so learners move from passive observation to active clinical reasoning. The second development is the emergence and validation of structured teaching models. Frameworks such as SNAPPS (Summarize, Narrow, Analyze, Probe, Plan, Select), a learner-centered model designed to develop clinical reasoning in outpatient settings, the One-Minute Preceptor (OMP), a teacher-centered model for efficient, time-constrained bedside teaching, and the Briefing-Intraoperative-Debriefing (BID) model, a process-centered framework for teaching in the operating room, offer systematic approaches that reduce extraneous cognitive load and ensure consistent attention to key teaching moments (10–12). Each model operationalizes constructivist principles in ways suited to different clinical settings, outpatient clinics, busy inpatient rounds, or the operating room. Third is the rapid advance of intelligent technologies. Generative AI large language models can now simulate consultant-level questioning, helping trainees prepare for unpredictable queries during rounds (13, 14). Immersive virtual reality (IVR), delivered through head-mounted displays, offers scalable, distributed training that demonstrates superior long-term knowledge retention compared with traditional methods (15). However, these technologies are not replacements for human teaching. They function best as augmentations that address specific gaps, pre-round preparation, post-round consolidation, and skills practice in low-stakes environments (16, 17).

This review synthesizes evidence across these three domains to examine how surgical teaching rounds might be redesigned. It first outlines the theoretical foundations drawn from constructivism and cognitive load theory. It compares three structured teaching models, SNAPPS, OMP, and BID, looking at their procedural steps, evidence base, and suitability for surgical settings. Review then examines emerging applications of generative AI and immersive VR, along with their limitations and ethical considerations. Building on this, we propose an integrated framework that treats theory as the foundation, structured models as the methodology, and technology as an empowering tool. The key research gaps are identified, and we conclude with implications for practice and future investigation.

## 2 Theoretical foundations: constructivism and cognitive load in surgical education

This is a narrative review. It integrates theoretical foundations, structured teaching models, and emerging technologies to propose a coherent framework for surgical ward round education. As a narrative review, it does not employ a systematic search strategy, predefined eligibility criteria, or formal risk-of-bias assessment. Instead, relevant literature was identified through targeted searches of PubMed, EMBase, Web of Science, and Scopus, supplemented by the authors’ domain expertise, with the aim of providing a broad conceptual synthesis and generating hypotheses for future research.

### 2.1 Constructivism: building knowledge through active engagement

At its core, constructivism rejects the idea that knowledge is simply transferred from teacher to learner. Instead, learners actively construct understanding by integrating new information with what they already know, in interaction with their environment (18). This stands in contrast to behaviorist approaches that focus on an observable stimulus–response pattern without accounting for internal cognitive processes. In health professions education, constructivism is now recognized as one of several foundational theoretical perspectives, alongside cognitive learning processes and social theories of learning (19, 20).

Two distinct strands of constructivism inform educational practice. Cognitive constructivism, rooted in the Piaget’s work, emphasizes individual knowledge construction through processes of assimilation and accommodation. When learners encounter information that conflicts with existing mental frameworks, they experience cognitive disequilibrium and reorganize their schemas to the restore coherence. In surgical education, this translates into encouraging learners to actively engage with clinical problems, formulate and test hypotheses, and refine their understanding through a repeated exposure to increasingly complex cases (21, 22).

The social constructivism, drawing on Vygotsky’s sociocultural theory (23), extends this view by emphasizing that learning is fundamentally social. Two concepts are particularly relevant to clinical teaching. The Zone of Proximal Development (ZPD) refers to the space between what a learner can do independently and what can be achieved with guidance (24). Scaffolding describes the temporary support that more knowledgeable others, attending the surgeons, senior residents, provide to help learners progress through the ZPD (25).

**Figure 1:**
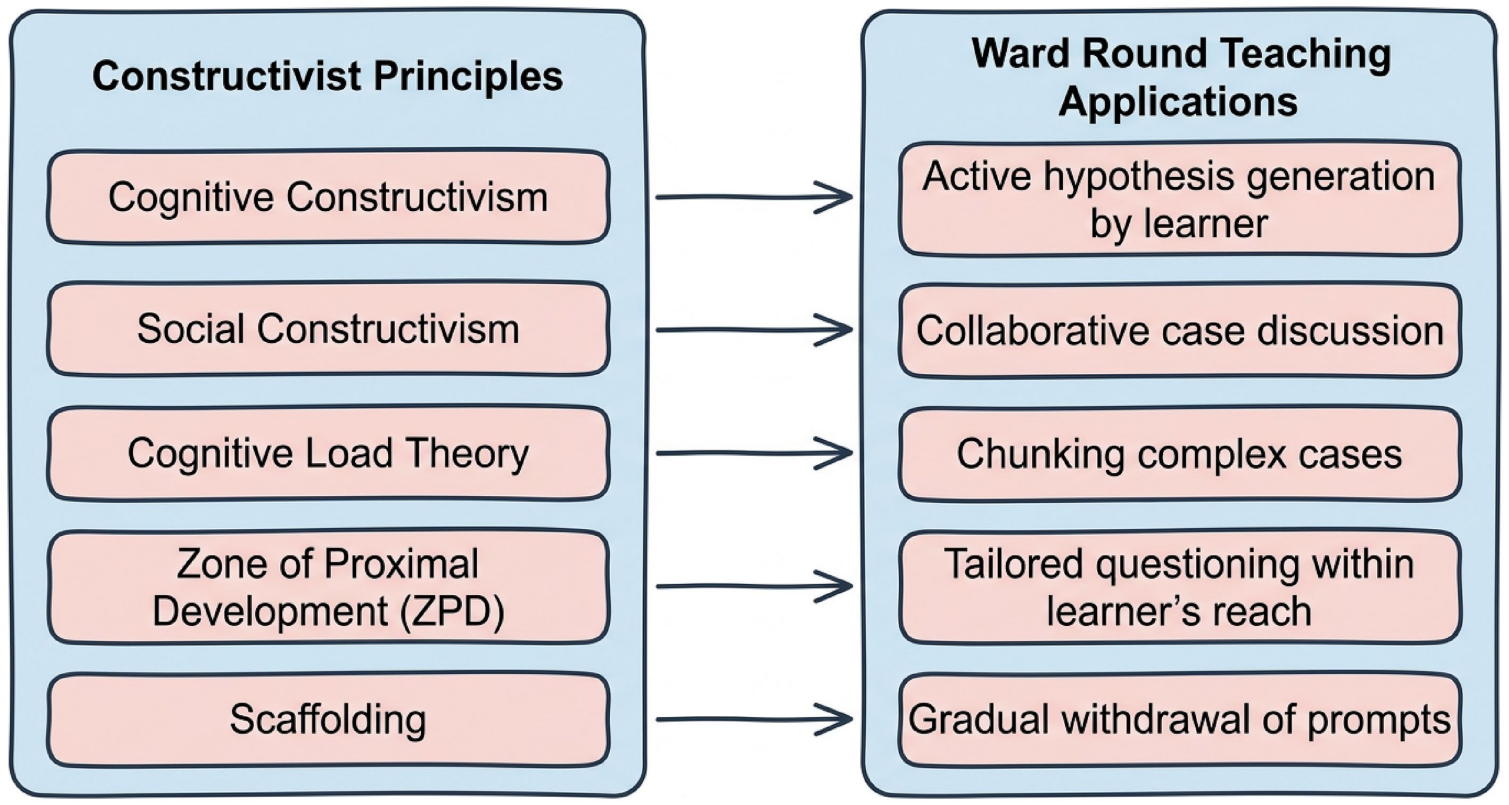
Integrated three-tier framework for surgical teaching round transformation.

Surgical ward rounds are, by their nature, social learning environments. Learners interact with attending surgeons, senior residents, nursing staff, and patients. Knowledge construction happens through observation, participation, questioning, feedback, and the collaborative problem-solving. Qualitative studies using constructivist grounded theory examined factors that shape when and how medical students speak up or remain silent in the operating room (26). Social dynamics, hierarchical expectations, and the implicit norms about appropriate behavior were found to powerfully shape learning opportunities (27, 28). Findings point to the importance of deliberately designing teaching interactions rather than leaving them to chance (Figure 1).

The concept of multi-pathway guided instruction emerges directly from constructivist thinking. Because learners construct knowledge through varied cognitive and social processes, the effective teaching must offer multiple routes for engagement, exploration, and consolidation (29). Surgical teacher’s role shifts from transmitter to facilitator: creating productive cognitive dissonance through the well-timed questions, providing the scaffolded support within each learner’s ZPD (30), fostering collaborative problem-solving, encouraging the metacognitive reflection, and tailoring guidance to individual needs (31, 32).

### 2.2 Cognitive load theory and its relevance to ward round teaching

The Cognitive Load Theory (CLT), offers a complementary lens for understanding the cognitive demands learners face during surgical rounds (33). CLT distinguishes three types of cognitive load. Intrinsic load reflects the inherent complexity of the material being learned. Extraneous load stems from suboptimal instructional design. Germane load is the cognitive effort devoted to building and automating schemas (34, 35). Key insight is that working memory capacity is limited. When intrinsic and extraneous loads exceed this capacity, learning suffers (36).

During surgical rounds, learners confront multiple simultaneous demands (37). They must attend to patient presentations, recall relevant pathophysiology, formulate diagnostic hypotheses, weigh management options, and respond to unpredictable questioning from senior clinicians, all while maintaining professional composure (38, 39). When teaching interactions are poorly structured, the extraneous load compounds an already high intrinsic load, overwhelming working memory and impairing retention (40).

Well-designed instructional approaches can reduce the extraneous load and the channel cognitive resources toward germane processes (41). Recent initiatives have explicitly integrated the CLT into clinical teaching design (42, 43). An obstetrics and gynecology teaching program incorporating simulation-based education under CLT principles demonstrated that managing all three load types, limiting intrinsic load through advance preparation, minimizing extraneous load through structured sessions, and promoting germane load through deliberate practice, optimized learning outcomes (44, 45). A lecture assessment tool rooted in CLT has also been developed and validated for medical education contexts, enabling systematic evaluation of instructional quality (46, 47). The structured teaching models SNAPPS, OMP, and BID can be understood as practical applications of CLT principles (48–50). Each model reduces extraneous load by providing a predictable framework for teaching interactions, freeing cognitive resources for the germane work of clinical reasoning and skill development.

## 3 Structured teaching models in surgical ward rounds

### 3.1 Overview

The structured teaching models organize clinical teaching around the defined steps, roles, and expectations. They provide cognitive scaffolding for both teachers and learners, reducing extraneous load and ensuring that key pedagogical elements, activating prior knowledge, probing reasoning, providing feedback, are not overlooked in the press of clinical work (51, 52).

A scoping review of intraoperative teaching methods identified multiple structured approaches applicable to surgical education, including the BID model and the STIC framework (Set, Target, Inspect, Close) (5). Among these, three models have accumulated the most substantial evidence base and demonstrated clear applicability to surgical contexts: SNAPPS, OMP, and the BID framework. Each offers distinct advantages and is suited to different teaching scenarios, learner levels, and clinical settings (Table 1).

**Table 1:** Comparative characteristics of structured teaching models.

Feature	SNAPPS (11)	OMP (136)	BID model (12, 137)
Primary orientation	Learner-centered	Teacher-centered	Process-centered
Number of steps	6 (Summarize, Narrow, Analyze, Probe, Plan, Select)	5 (Get a commitment, Probe for supporting evidence, Teach general principles, Reinforce what was done well, Correct mistakes)	3 (Briefing, Intraoperative teaching, Debriefing)
Optimal context	Outpatient clinics, senior trainees, elective rotations	High-volume ward rounds, junior trainees, bedside teaching	Operating room, intraoperative teaching
Core mechanism	Active hypothesis-driven reasoning by learner	Efficient micro-skills for time-constrained teaching	Structured pre-intra-post-operative learning loop
Key advantage	Superior clinical reasoning development	High learner satisfaction, efficient feedback	Aligned with competency-based assessment
Primary limitation	Time-intensive; requires baseline learner competence	Limited depth for advanced reasoning	Requires faculty preparation and dedicated time
Key evidence (5)	SNAPPS superior to OMP in case presentation quality and differential diagnoses in simulated and real settings	OMP received higher satisfaction scores than SNAPPS; both superior to traditional teaching	Encompasses perioperative teaching time points; compatible with competency assessments
Recommended learner level	Senior medical students, senior residents	Junior medical students, junior residents, interns	Surgical residents (all levels)

### 3.2 SNAPPS: learner-centered clinical reasoning development

SNAPPS is a learner-centered, six-step framework designed to promote active engagement in clinical reasoning and self-directed learning (11). Acronym stands for: Summarize relevant patient history and physical examination findings; Narrow the differential diagnosis to two or three most likely possibilities; Analyze the differential by comparing and contrasting diagnostic possibilities; Probe the preceptor by asking questions about uncertainties or knowledge gaps; Plan management for the patient’s medical issues; and Select a case-related issue for self-directed learning (53, 54).

**Figure 2:**
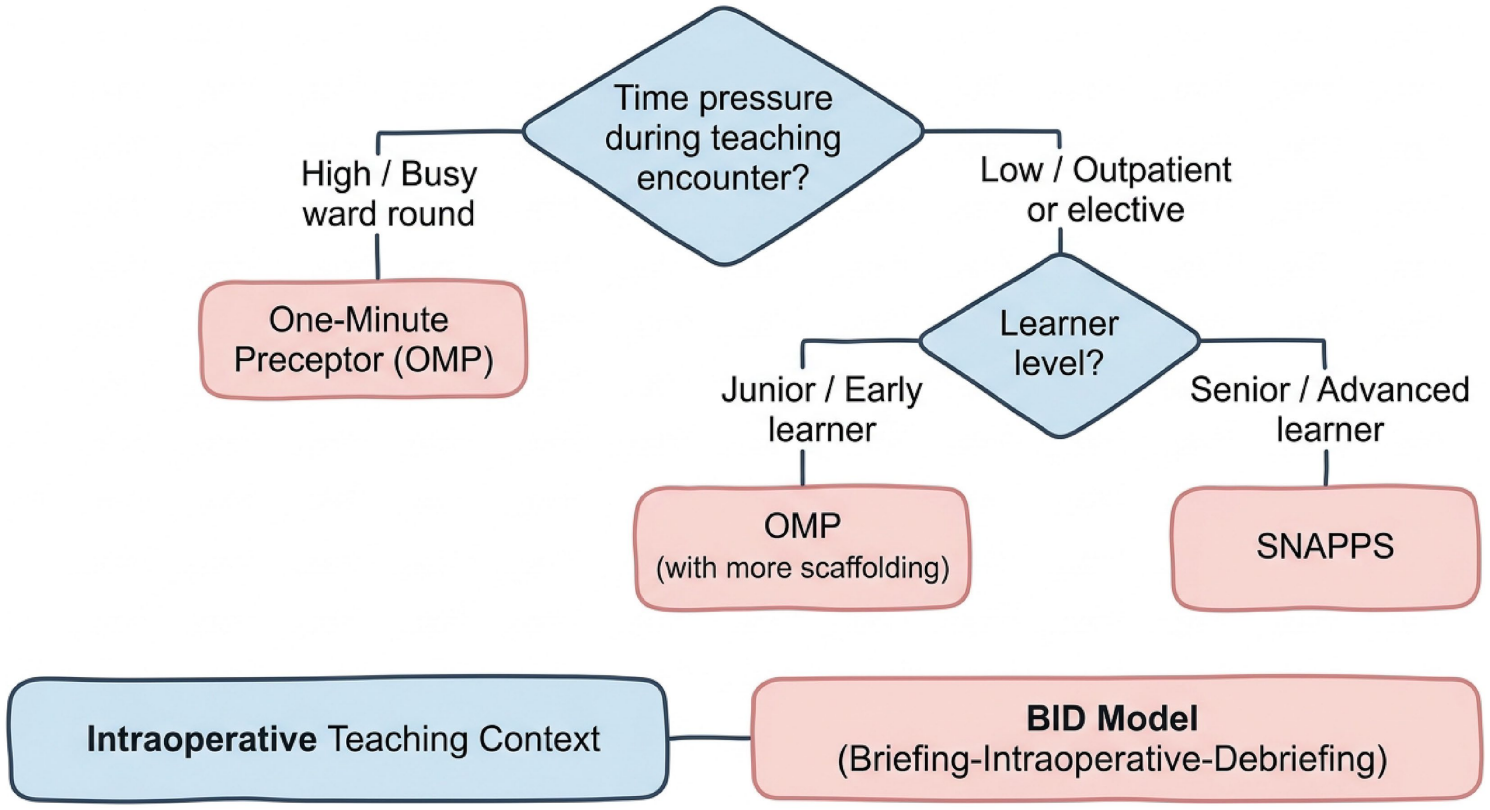
Three-phase workflow for technology-enhanced surgical ward rounds.

The framework embodies constructivist principles in several ways. Narrow and Analyze steps compel learners to engage in hypothesis-driven reasoning rather than passive recitation (55). Probe step explicitly encourages articulation of uncertainties, normalizing the cognitive disequilibrium essential for learning. The Select step promotes metacognitive awareness by requiring learners to identify and pursue individualized learning objectives (Figure 2) (56, 57).

The effectiveness of SNAPPS has been evaluated in multiple randomized controlled trials. Grunewald et al. (5) randomized 256 medical students during their pediatrics clerkship into three arms: SNAPPS (*n* = 87), OMP (*n* = 83), and traditional teaching control (*n* = 86). Both intervention arms performed significantly better than controls across multiple outcomes. Notably, the SNAPPS produced superior results compared with OMP in the simulated environment for discussion time, the case presentation quality score, student performance score, and number of differential diagnoses presented and justified. In real clinical settings, SNAPPS remained superior to OMP for case presentation quality score (58–60).

A systematic review with meta-analysis of randomized controlled trials examining SNAPPS in clinical reasoning teaching found that while evidence certainty was low to moderate, SNAPPS may increase the odds of trainees initiating a management plan and seeking clarification. The review noted that SNAPPS appears more beneficial when used by residents than by medical students (6, 61).

Fagundes et al. (60) conducted a randomized controlled trial (RCT) comparing SNAPPS and OMP in 60 medical students at the start of their fifth year. Both methods equally promoted expression of clinical reasoning, but students in SNAPPS group expressed significantly more questions and uncertainties and more often took initiative to present and justify the most likely diagnosis, differential diagnosis, and management plan, without extending session length (62, 63).

The SNAPPS is particularly suited to surgical educational contexts where clinical reasoning development is paramount, outpatient clinics and post-operative patient evaluation, where time constraints are less acute than in intraoperative settings (10, 64). Model’s emphasis on differential diagnosis generation and analysis aligns well with the cognitive demands of surgical practice.

Limitations include the time required to complete all six steps, which may not be feasible during high-volume ward rounds or emergency consultations. The learner-centered orientation assumes a baseline level of clinical knowledge and confidence that junior learners may not possess, potentially generating anxiety rather than productive engagement (65). Implementation in surgical residency programs may require adaptation, abbreviated versions for time-pressured contexts, or graduated introduction of steps as learner competence increases (59).

### 3.3 OMP: efficiency-oriented clinical teaching

OMP model represents an efficiency-oriented, teacher-centered framework designed to maximize educational impact within the constraints of busy clinical practice (66). The model comprises five sequential microskills: Get a commitment, elicit learner’s opinion or diagnosis; Probe for supporting evidence, ask the learner to justify their reasoning; Teach general principles, provide a focused teaching point applicable beyond the specific case; Reinforce what was done well, offer positive feedback on specific aspects of performance; and Correct mistakes, provide constructive feedback on errors or omissions (67, 68).

The OMP framework operationalizes constructivist principles through a teacher-facilitated (69), scaffolded approach. Commitment and probing steps compel learners to externalize their cognitive processes, making reasoning visible for both teacher assessment and learner reflection. The teach general principles step promotes transfer of learning beyond the immediate case (70). The feedback components provide scaffolding for skill refinement (71).

Woodward et al. (67) evaluated OMP implementation in surgical training by incorporating a one-hour, resident-led OMP session into surgical intern orientation. Comparing medical student evaluations of residents who received OMP training (2023 cohort) with those who did not (2022 cohort), the intervention was associated with higher frequency of weekly study sessions (94% versus 62%, *p* = 0.022). Qualitative analysis revealed distinct themes: the 2022 cohort emphasized establishing psychological safety, while 2023 cohort prioritized intentional medical student teaching and modeling efficient, competent, and empathetic patient care (72, 73).

The Grunewald RCT revealed important distinctions between OMP and SNAPPS. While both intervention arms outperformed traditional teaching, OMP received significantly higher satisfaction ratings compared with SNAPPS, despite SNAPPS demonstrating superiority in clinical reasoning outcomes (5, 10, 74). This suggests learner satisfaction and cognitive skill development may not always align, and different models may serve different educational objectives (Table 2).

**Table 2:** Detailed procedural steps of SNAPPS and OMP models.

Step	SNAPPS (11, 138)	OMP (139, 140)
1	Summarize relevant patient history and physical examination findings	Get a commitment — elicit the learner’s opinion or diagnosis
2	Narrow the differential diagnosis to two or three most likely possibilities	Probe for supporting evidence — ask the learner to justify their reasoning
3	Analyze the differential by comparing and contrasting diagnostic possibilities	Teach general principles — provide a focused teaching point applicable beyond the case
4	Probe the preceptor by asking questions about uncertainties or management dilemmas	Reinforce what was done well — offer positive feedback on specific aspects
5	Plan management for the patient’s medical issues	Correct mistakes — provide constructive feedback on errors or omissions
6	Select a case-related issue for self-directed learning	—

The comparative evidence reveals complementary rather than competing roles. OMP’s teacher-centered, efficiency-oriented design suits high-volume clinical settings, bedside teaching during rapid ward rounds, and situations where learners possess the limited baseline knowledge (10). The SNAPPS’s learner-centered orientation is more appropriate for outpatient clinic teaching, elective rotations, and senior residents capable of engaging in advanced clinical reasoning. Selection should be guided by time availability, learner level, specific objectives, and the nature of the clinical encounter (75, 76).

### 3.4 BID model: structured framework for intraoperative teaching

The BID model was specifically developed for surgical teaching in the operating room. Based on a guided discovery learning model, it organizes the teaching encounter into three phases (12). The Briefing phase occurs preoperatively and establishes clear expectations, identifies targeted skills or knowledge to be addressed, and prepares both teacher and learner. Intraoperative teaching segment consists of immediate feedback and guidance directed by predetermined learning objectives, with the attending surgeon providing scaffolded instruction, modeling expert performance, and offering real-time corrections within the learner’s ZPD (77). The debriefing phase solidifies learning and enables metacognitive processing, identification of areas for the improvement, and integration of procedural knowledge with conceptual understanding (48, 78).

A scoping review of intraoperative teaching methods identified BID model as a comprehensive framework that encompasses perioperative teaching time points/suggests universal organizational approach compatible with competency-based assessment requirements (79). The BID model directly addresses documented deficiencies in traditional intraoperative teaching. Only a minority of resident report that faculty help identify educational goals preoperatively or discuss areas for improvement postoperatively. The BID framework targets these gaps by mandating explicit goal-setting in the briefing phase and systematic feedback in the debriefing phase (80, 81).

Implementation challenges include the model’s dependence on faculty preparation and deliberate time allocation for briefing and debriefing, resources often constrained in high-volume surgical practices (82). Faculty must be comfortable with structured teaching methodologies that differ from traditional apprenticeship-style instruction. Faculty development initiatives are essential: surgeons need training in formulating specific, achievable learning objectives (83); providing constructive intraoperative feedback without disrupting surgical flow (84); and conducting effective debriefing sessions that promote reflection without inducing defensive responses.

## 4 Converging pathways: intelligent technologies in surgical education

### 4.1 Generative AI in ward round preparation and consolidation

Recent advances in generative AI large language models have introduced novel possibilities for enhancing surgical ward round education. A 2025 study evaluated ChatGPT-4.5 and Gemini 2.0 across hypothetical plastic and reconstructive surgery scenarios including flexor tenosynovitis, deep inferior epigastric perforator (DIEP) flap monitoring, acute burns, and abscess management (1). ChatGPT-4.5 demonstrated strong capacity to anticipate the consultant-style questions and deliver concise, the accurate responses, consistently outperforming Gemini 2.0 in expert Likert ratings for accuracy, clarity, and educational value (85–87).

The educational applications span pre-round, intra-round, and post-round phases. AI can simulate consultant-level questioning based on anticipated case presentations, enabling trainees to practice clinical reasoning and communication skills in a low-stakes environment (88). Moreover, the AI systems can generate tailored summaries of the key learning points, create practice questions, and produce management algorithms that reinforce clinical reasoning patterns (89, 90). Limitations are significant. The study noted occasional inaccuracies in AI-generated content, risks of learner over-reliance, and privacy considerations when AI systems are used in clinical contexts (91). The potential erosion of critical thinking skills through excessive AI dependence requires careful attention (92, 93).

Ethical considerations encompass privacy, bias, informed consent, and algorithmic transparency. A scoping review mapped the integration of AI in undergraduate medical education, identifying ethical training, collaborative learning, and digital competence as essential elements, with emphasis on transversal skills that position AI as a tool rather than a standalone subject (94, 95). Algorithmic bias is a particular concern: AI systems trained on datasets that underrepresent certain populations or perpetuate existing healthcare disparities may reinforce rather than remediate biases in clinical reasoning. Governance and regulatory frameworks remain underdeveloped. A 2025 review in NPJ Digital Medicine identified pedagogical considerations, emerging roles for AI, and trustworthiness as key domains requiring regulatory attention (96).

### 4.2 Immersive virtual reality in surgical skills training

IVR based on head-mounted displays has emerged as a promising educational tool. A 2025 systematic review found that IVR is particularly effective in practice-based medical education, with the superior long-term knowledge retention and knowledge enhancement compared with traditional methods. The findings suggest effectiveness in clinical surgery training, anatomy education, medical skill development, and nursing education (15).

In surgical contexts, the VR applications have demonstrated utility for robotic-assisted surgery training. A feasibility study comparing head-mounted VR simulation with conventional console-based simulation found that both modalities produced significant skill improvements, with the VR training group requiring less time (mean difference 39 ± 9.01 min, *p* = 0.004) and fewer attempts (mean difference 8 ± 2.2, *p* = 0.009) to reach proficiency benchmarks (97).

The application of VR to ward round teaching represents an emerging frontier. Immersive simulations could enable learners to practice clinical reasoning, communication skills, and team-based decision-making in realistic yet controlled settings. However, substantial practical barriers remain (98): equipment costs, technical infrastructure requirements, and faculty digital literacy gaps. The systematic review emphasized the need for comprehensive evaluation incorporating perspectives from teachers and consideration of the “dark side” of VR, including technology-induced stress and time requirements (99, 100).

### 4.3 Augmentation, not replacement

Evidence across AI and VR applications converges on a consistent theme: technology functions most effectively as an augmentation to human instruction, not a replacement for it. The Bacchi study’s conclusion that generative AI shows promise as a supplementary tool in surgical ward-round education accurately captures the appropriate positioning of these technologies (101, 102).

Several principles for human-technology integration emerge. Technology should address specific, identified educational gaps. Faculty development is essential for effective integration. Learners must develop critical awareness of technology limitations (Table 3). The governance frameworks must evolve in parallel with technological capabilities to ensure the ethical, equitable, and educationally sound implementation (96, 103).

**Table 3:** Applications and limitations of intelligent technologies in surgical education.

Technology	Educational application	Key advantages	Limitations and challenges
Generative AI (LLMs)	Pre-round simulated questioning; post-round tailored summaries; management algorithm generation	Simulates consultant-level questioning; reduces trainee anxiety; enables personalized learning (141, 142)	Occasional factual inaccuracies; risk of learner over-reliance; privacy considerations; algorithmic bias (143, 144)
AI-enhanced feedback	Real-time assessment of case presentation structure; objective feedback on content completeness	Consistent, scalable feedback; reduces faculty assessment burden (145, 146)	May not capture nuanced clinical judgment; requires validation before clinical deployment (147, 148)
Head-mounted VR	Immersive surgical skills training; robotic surgery simulation	Shorter time and fewer attempts to reach proficiency benchmarks; equivalent skill acquisition to console-based training (149, 150)	Non-inferiority not fully established; equipment costs remain significant; faculty digital literacy gaps (151)
Remote synchronized VR	Multi-center surgical teaching workshops; 3D anatomical exploration	Overcomes geographic barriers; significant improvements in procedural confidence (152, 153)	Requires robust technical infrastructure; variable internet connectivity across sites (154, 155)
Multi-agent AI framework	Interactive neurosurgical vignettes; virtual clinical conversations	Engaging evaluation method for LLMs; specialty training tool (156, 157)	Early-stage development; requires validation in diverse subspecialties (158)

This positioning of technology as augmentation rather than replacement aligns with broader trends across science education. A growing body of literature has examined the current status and future prospects of AI integration in science training. Recent systematic reviews document that AI applications, ranging from intelligent tutoring systems to generative AI tools such as ChatGPT, have demonstrated favorable impacts on student performance, motivation, and engagement, particularly through personalized learning and the development of self-regulated learning skills (104). Bibliometric analyses of over 3,200 studies (2015–2024) identified four dominant thematic clusters: intelligent tutoring and adaptive learning, virtual and remote laboratories, generative AI and ethics, and teacher professional development, with meta-analytic evidence confirming a significant overall positive effect on learning outcomes (Hedges’ *g* = 0.63), particularly in higher education (*g* = 0.75) (105). However, these reviews also consistently identify critical challenges, including concerns about algorithmic bias, information reliability, privacy, teachers’ preparedness, and the need for comprehensive professional development (106). A key cross-cutting finding is that AI functions most effectively when integrated within established pedagogical frameworks, such as constructivist and active learning approaches, rather than deployed as a standalone solution (107). These insights reinforce the central argument of our review: structured teaching models and constructivist principles provide the essential pedagogical infrastructure for meaningful technology integration, and advancing the field requires coordinated research with standardized outcomes and longitudinal follow-up.

In the proposed framework, the roles of AI and IVR are delineated across the three phases of ward-round teaching. Before rounds, generative AI enables learners to rehearse case presentations and anticipate consultant-style questions in a low-stakes environment, reducing cognitive load during actual patient encounters. During rounds, the selected structured teaching model—SNAPPS, OMP, or BID—remains the central pedagogical mechanism, with the attending surgeon providing scaffolded guidance; technology serves as a preparatory and consolidative adjunct rather than an intra-round replacement. After rounds, AI generates personalized summaries and practice questions targeting individual knowledge gaps, while IVR offers immersive scenarios for deliberate practice of clinical communication and reasoning skills encountered during the round. This three-phase deployment ensures that technology augments the human teaching interaction precisely where it is most needed—before and after the face-to-face clinical encounter—while preserving the irreplaceable value of expert modeling and real-time feedback during the round itself.

## 5 Toward an integrated framework

### 5.1 Synthesis: constructivism, structured models, technology

**Figure 3:**
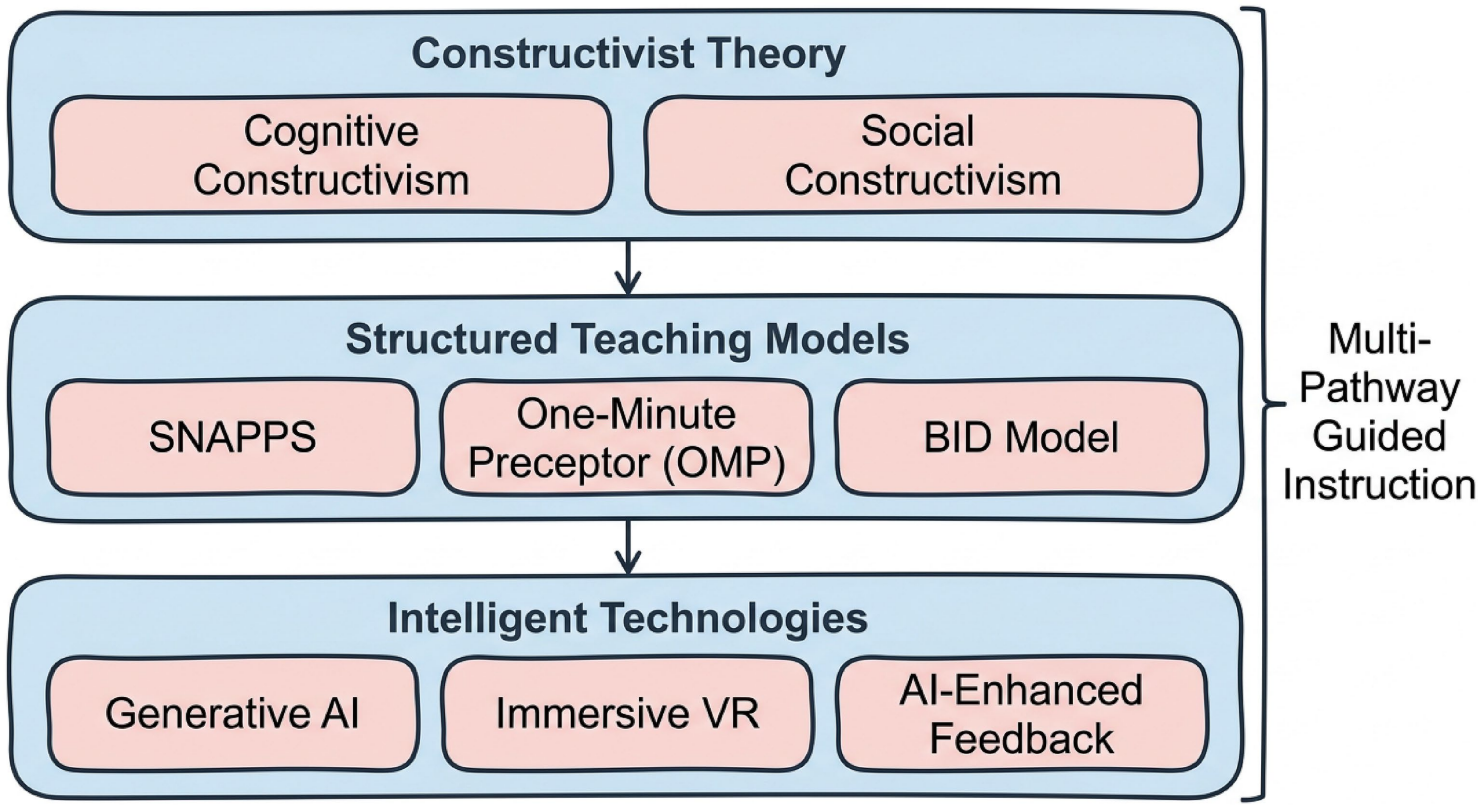
Decision algorithm for structured teaching model selection in surgical ward rounds.

The evidence reviewed reveals a coherent, synergistic relationship among the three domains. The constructivism provides the theoretical foundation, explaining why learner-centered, multi-pathway approaches enhance educational outcomes and offering principles for instructional design (108). Structured teaching models operationalize these principles within the practical constraints of clinical environments, translating abstract theory into concrete, replicable pedagogical procedures (109, 110). Emerging technologies expand the reach and capabilities of both theory and models, enabling new forms of learner engagement, personalized instruction, and distributed training (111). Relationship suggests an integrated paradigm: constructivist theory informs the selection and adaptation of structured teaching models, while intelligent technologies empower the implementation and scaling of these models across diverse educational contexts (Figure 3).

### 5.2 Proposed integrated model for surgical teaching rounds

Drawing on the available evidence, which remains preliminary in several areas, an integrated model can be articulated that incorporates three complementary components. Faculty should select structured teaching models based on contextual factors including time availability, learner level, specific learning objectives, and the nature of the clinical encounter. For high-volume ward rounds with junior learners, the OMP model’s efficiency and structured feedback components may be optimal, although evidence supporting this preference derives largely from observational studies and learner satisfaction data rather than comparative effectiveness trials (112, 113). F For outpatient clinic teaching with senior residents, SNAPPS’s emphasis on self-directed learning and advanced clinical reasoning may be preferable, though the supporting evidence consists of a small number of RCTs with low-to-moderate certainty and no outcomes at Kirkpatrick Levels 3 or 4 (114). For intraoperative teaching, the BID framework offers a theoretically grounded structure aligned with competency-based assessment, but its empirical evaluation remains limited, and its effectiveness relative to alternative intraoperative teaching models has not been established through rigorous comparative research (48).

Regardless of the model selected, implementation should embody constructivist principles of active learning, scaffolded support, social interaction, and metacognitive reflection. This requires faculty development in the theoretical underpinnings of their chosen models, not merely procedural familiarity with steps. Faculty should understand why particular teaching behaviors promote learning and be able to adapt their approach in response to individual learner needs (115, 116).

**Figure 4:**
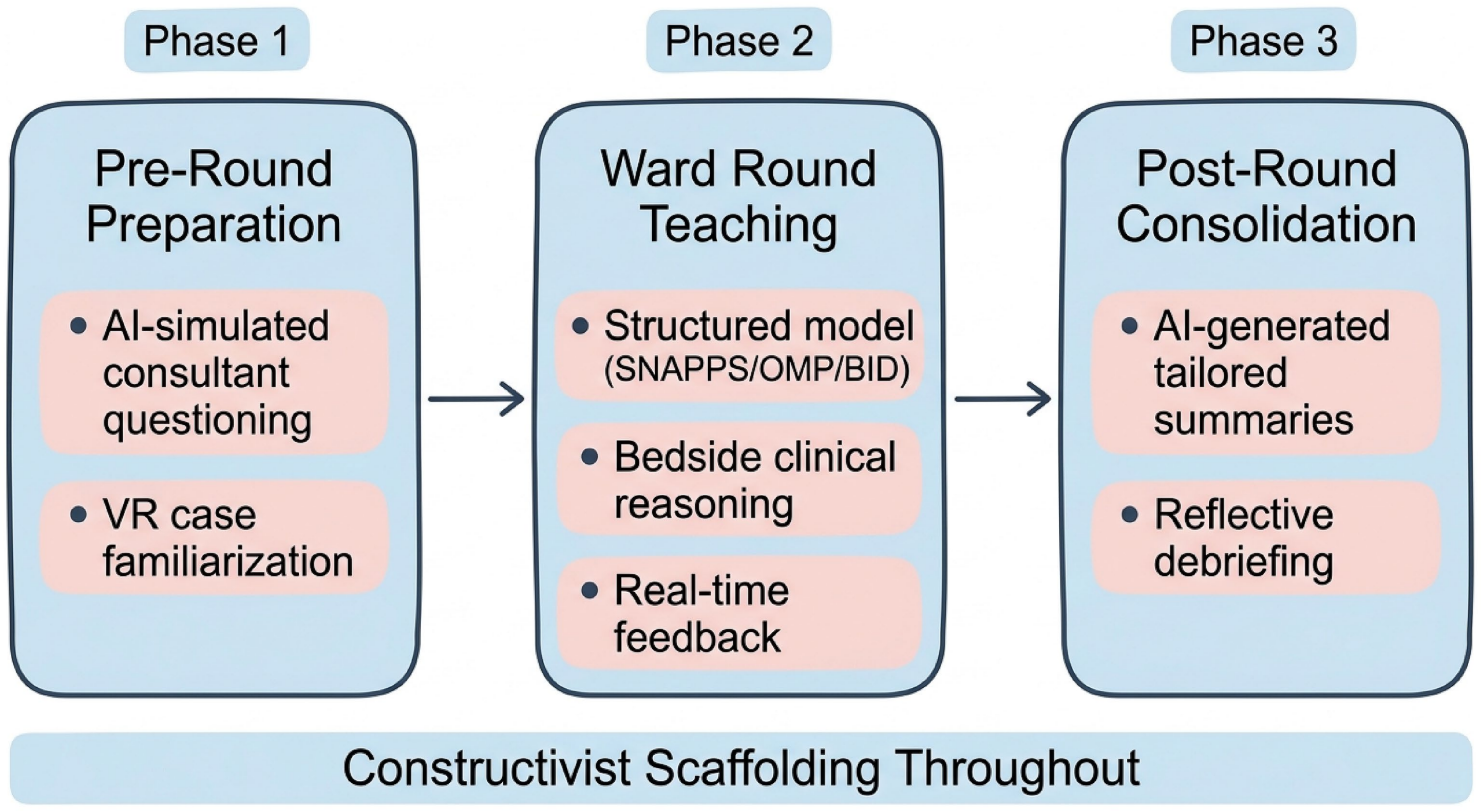
Mapping constructivist principles to ward round teaching applications.

Intelligent technologies should be deployed to address specific gaps in current educational practice. Generative AI, evaluated primarily through proof-of-concept studies using hypothetical scenarios, shows potential to support pre-round preparation and post-round consolidation, which may reduce cognitive load during actual patient encounters (117, 118). VR simulations may provide opportunities for deliberate practice of clinical reasoning and communication skills in low-stakes environments, with preliminary evidence suggesting possible benefits for skill development, though the evidence base for long-term knowledge retention remains limited (119). Technology integration should be guided by the principle of augmentation—enhancing human teaching capacity rather than substituting for it—and its effectiveness should be evaluated through rigorous multi-center studies with standardized outcomes before widespread implementation can be recommended (Figure 4).

### 5.3 Implications for curriculum design and faculty development

Implementation of this integrated framework requires systematic attention to curriculum design and faculty development. The surgical training programs should incorporate explicit instruction in structured teaching models, not only for attending surgeons but also for the senior residents who function as near-peer teachers (120, 121). Faculty development programs should address the ability to formulate learner-appropriate objectives, skill in providing specific actionable feedback within time-constrained encounters, proficiency in multiple teaching models, understanding of cognitive load principles, familiarity with educational technologies, and awareness of ethical considerations in technology-enhanced education (122). Assessment of the teaching quality should incorporate validated instruments aligned with the integrated framework, evaluating not only frequency of teaching interactions but also their quality, adherence to structured model elements, and alignment with constructivist principles (123, 124).

## 6 Research gaps and future directions

### 6.1 Methodological limitations in current evidence

Several methodological limitations characterize the current evidence base. Outcome measures are highly heterogeneous across studies, complicating comparison and meta-analytic synthesis (125). Some studies assess the learner satisfaction, others evaluate knowledge acquisition, and still others measure clinical reasoning performance, but the relationships among these outcomes remain poorly characterized (126, 127). The development of a core outcome set for surgical teaching interventions would significantly advance the field (Table 4).

**Table 4:** Summary of key studies on structured teaching models and technology-enhanced surgical education.

Study	Design	Specialty/setting	Participants (sample size)	Comparator	Primary outcome	Reported statistics	Limitations
Grunewald et al. (5)	RCT	Pediatrics clerkship	256 medical students (SNAPPS *n* = 87; OMP *n* = 83; control *n* = 86)	OMP vs. traditional teaching	Case presentation quality; number of differential diagnoses	Both interventions superior to control; SNAPPS superior to OMP for case presentation quality score	Single-institution; no Kirkpatrick Level 3/4 outcomes; short follow-up
Fagundes et al. (60)	RCT	General medicine (controlled setting)	60 fifth-year medical students	OMP	Expression of clinical reasoning; number of questions and uncertainties expressed	SNAPPS group expressed more questions and uncertainties; no difference in session length	Small sample size; simulated rather than real clinical setting
Flores-Cohaila et al. (6)	Systematic review with meta-analysis	Clinical reasoning teaching (various)	5 RCTs (total n varied)	Traditional teaching or OMP	Clinical reasoning outcomes; discussion length; differential diagnoses	Low-to-moderate certainty evidence; SNAPPS may increase discussion length and expression of uncertainties	Only five included RCTs; high heterogeneity; no patient-level outcomes
Woodward et al. (67)	Cohort comparison	General surgery internship	Surgical interns (two cohorts: 2022 vs. 2023)	No OMP training (historical control)	Frequency of weekly study sessions; qualitative teaching themes	Higher study session frequency with OMP training (94% vs. 62%, *p* = 0.022)	Single-institution; non-randomized; possible cohort effects
Schulze et al. (159)	Scoping review	Surgical resident education	66 studies	Various intraoperative teaching frameworks	Identification of teaching frameworks	BID model encompasses perioperative teaching time points; compatible with competency assessments	Scoping review design; no quantitative synthesis
Bacchi et al. (1)	Qualitative/exploratory	Plastic and reconstructive surgery (hypothetical scenarios)	Hypothetical scenarios (ChatGPT-4.5 vs. Gemini 2.0)	Gemini 2.0	Accuracy, clarity, educational value (Likert ratings)	ChatGPT-4.5 consistently outperformed Gemini 2.0	Hypothetical scenarios; no real clinical application; small number of raters
Kawashima et al. (97)	RCT with crossover	Robotic surgery training	14 participants	Console-based VR simulation	Time and number of attempts to reach proficiency benchmark	VR group required less time (mean difference 39 ± 9.01 min, *p* = 0.004) and fewer attempts (mean difference 8 ± 2.2, *p* = 0.009)	Very small sample size; non-inferiority not established
Zhang et al. (15)	Systematic review	Medical education (various)	36 studies	Traditional methods	Long-term knowledge retention; knowledge enhancement	IVR particularly effective in practice-based education; superior long-term knowledge retention	Limited number of studies; heterogeneity in VR modalities; follow-up duration variable

The predominance of single-institution studies with small sample sizes limits generalizability. The multi-center trials with adequate statistical power are needed to establish effectiveness across diverse institutional contexts, learner populations, and surgical specialties (128, 129). The duration of follow-up in most studies is insufficient to assess durability of educational effects. Longitudinal studies tracking learner outcomes over months to years would provide critical evidence regarding long-term impact on clinical competence and career development (130). A detailed methodological summary of the key studies cited in this review, including study design, sample size, primary outcomes, reported statistics, and limitations, is provided in Table 4.

### 6.2 Under-explored areas

Several important domains remain under-explored. The role of learner characteristics, including baseline knowledge, learning style preferences, anxiety, and the self-efficacy, in moderating the effectiveness of different structured teaching models warrants systematic investigation. Interaction between teaching model characteristics and specific surgical subspecialty contexts has received minimal attention.

Optimal integration of intelligent technologies with structured teaching frameworks represents a particularly important frontier. While individual studies have examined AI or VR applications in isolation, research systematically investigating synergistic effects, whether AI-generated feedback enhances SNAPPS or OMP implementation, or whether the VR ward round simulations improve learner readiness for actual clinical encounters, is largely absent.

### 6.3 Recommendations for future research

Advancing the field requires coordinated, multi-center research initiatives with standardized methodological approaches. Priorities include development and validation of a core outcome set for surgical teaching interventions, encompassing cognitive (clinical reasoning, knowledge retention), behavioral (case presentation quality, differential diagnosis generation), and affective (the learner satisfaction, psychological safety) domains (131, 132). The multi-center randomized trials should directly compare the effectiveness of the different structured teaching models in specific surgical educational contexts (133). Implementation science approaches should identify barriers and facilitators to adoption of structured teaching models in surgical training programs. Systematic technology integration studies should investigate how AI and VR can be optimally combined with structured teaching frameworks, including the effects on learner cognitive load, faculty teaching efficiency, and educational equity (134, 135).

To evaluate the value of the proposed integrated methodology, future studies should consider measurable outcomes across three domains aligned with the framework’s components. For structured teaching models, outcomes may include: number and quality of differential diagnoses generated; case presentation completeness scores using validated rubrics; and learner expression of uncertainties and self-directed learning questions. For technology integration, outcomes may include: pre-round preparation time and quality; post-round knowledge retention measured through spaced testing; and learner self-efficacy and anxiety ratings before and after AI-assisted preparation. At the program level, implementation outcomes—such as faculty adoption rates, fidelity to structured model steps, and learner satisfaction—should complement cognitive and behavioral measures. Standardizing these outcomes across studies, as recommended in the core outcome set proposed earlier, would substantially strengthen the evidence base and enable meaningful comparisons across interventions.

## 7 Conclusion

Surgical ward rounds remain a cornerstone of clinical education but face persistent challenges from time compression, increasing clinical complexity, and heightened expectations for educational accountability. This review has synthesized evidence across the three interconnected domains: constructivist learning theories, structured teaching models, and emerging intelligent technologies.

Constructivism, encompassing both cognitive and social traditions, provides a robust theoretical foundation for learner-centered, multi-pathway guided instruction. Cognitive load theory offers complementary insights into managing the cognitive demands of clinical learning environments. Together, these frameworks justify moving beyond the traditional apprenticeship models toward systematic, intentionally designed approaches.

The structured teaching models, SNAPPS, OMP, and the BID framework, the operationalize constructivist principles through defined procedural steps and articulated teacher-learner roles. Preliminary comparative evidence suggests that SNAPPS may be more effective for developing clinical reasoning skills and improving case presentation quality, though the available systematic review includes only five RCTs with low-to-moderate certainty and no outcomes at the level of clinical practice or patient impact. OMP appears to offer greater efficiency and higher learner satisfaction in time-constrained environments. The BID model provides a theoretically grounded framework for intraoperative teaching that addresses documented deficiencies in traditional surgical instruction, although its empirical evaluation remains limited.

Emerging technologies, generative AI and immersive VR, present unprecedented opportunities. Generative AI, evaluated primarily through proof-of-concept studies using hypothetical scenarios, shows potential to simulate consultant-level questioning and support pre-round preparation and post-round consolidation. IVR may offer immersive, distributed training, with preliminary evidence suggesting possible benefits for skill development and knowledge retention. However, these technologies also introduce ethical considerations, including algorithmic bias, privacy concerns, and risks of over-reliance, that require careful attention and robust governance frameworks.

An integrated paradigm, constructivist theory informing structured model selection, with intelligent technologies empowering implementation, is proposed to guide future development of surgical teaching rounds. This paradigm emphasizes the complementary, mutually reinforcing relationships among theoretical foundations, pedagogical methodologies, and technological tools. Implementation requires systematic attention to curriculum design, faculty development, and ongoing assessment of educational outcomes.

Field now needs coordinated, multi-center research initiatives with standardized methodological approaches. Development of core outcome sets, comparative effectiveness trials, implementation science studies, and systematic technology integration research will collectively advance surgical education toward the goal of teaching rounds that optimize learner development while maintaining excellence in patient care.
